# Regulation of nestin expression by thrombin and cell density in cultures of bone mesenchymal stem cells and radial glial cells

**DOI:** 10.1186/1471-2202-8-104

**Published:** 2007-11-30

**Authors:** Franz Wautier, Sabine Wislet-Gendebien, Grazyna Chanas, Bernard Rogister, Pierre Leprince

**Affiliations:** 1 Center for Cellular and Molecular Neurobiology, University of Liège, CHU B36, Avenue de l'Hôpital, 1, B-4000 Liège, Belgium; 2 Department of Neurology, University of Liège, CHU B35, B-4000 Liège, Belgium; 3 Department of Biochemistry and Cell Physiology, University of Liège, CHU B35, B-4000 Liège, Belgium

## Abstract

**Background:**

Bone marrow stromal cells and radial glia are two stem cell types with neural phenotypic plasticity. Bone marrow mesenchymal stem cells can differentiate into osteocytes, chondrocytes and adipocytes, but can also differentiate into non-mesenchymal cell, i.e. neural cells in appropriate *in vivo *and *in vitro *experimental conditions. Likewise, radial glial cells are the progenitors of many neurons in the developing cortex, but can also generate astrocytes. Both cell types express nestin, an intermediate filament protein which is the hallmark of neural precursors.

**Results:**

In this study, we demonstrate that thrombin, a multifunctional serine protease, stimulates the growth of radial glial cells (RG) and mesenchymal stem cells (MSCs) in a dose-dependent manner. In RG, the mitogenic effect of thrombin is correlated with increased expression of nestin but in MSCs, this mitogenic effect is associated with nestin down-regulation. Both cell types express the PAR-1 type receptor for Thrombin and the effect of Thrombin on both cell types can be mimicked by its analogue TRAP-6 activating specifically this receptor subtype or by serum which contains various amount of thrombin. Moreover, we also demonstrate that serum deprivation-induced expression of nestin in MSCs is inhibited by high cell density (> 50,000 cells/cm2).

**Conclusion:**

This work shows that thrombin stimulates the growth of both RG and MSCs and that nestin expression by MSCs and RG is regulated in opposite manner by thrombin *in vitro*. Thrombin effect is thus associated in both cell types with a proliferating, undifferentiated state but in RG this involves the induction of nestin expression, a marker of immaturity for neural progenitors. In MSCs however, nestin expression, as it corresponds to a progression from the mesenchymal "undifferentiated", proliferating phenotype toward acquisition of a neural fate, is inhibited by the mitogenic signal.

## Background

Stem cells have the capacity to self-replicate or to produce progeny of one or several specific differentiated cell types. There are two types of stem cells: embryonic and somatic stem cells, which are found in foetal and adult tissue respectively. While they are generally committed to a particular tissue fate, somatic stem cells can alternatively generate cells belonging to different tissue types when they are exposed to specific cues produced by a different tissue. High hopes rest on the future application of stem cells in cellular therapies, including those of the central nervous system disorders. For this reason, the mechanisms that regulate the acquisition of a neural fate by somatic stem cells are under intensive study. In this work, we will extend our investigations of the regulation of the expression of nestin, a neural stem cell marker, by mesenchymal stem cells (MSCs) and compare it to the corresponding process occurring in radial glial cells (RG), a major form of neural stem cell in mammalian central nervous system (CNS) development.

MSCs can differentiate into many types of mesenchymal phenotypes, i.e. osteocytes, chondrocytes and adipocytes, and they can also differentiate into non-mesenchymal cell, i.e. neural cells, when grafted in the lesioned central nervous system [1,2] or when grown *in vitro *together with neural cells [3-5]. In the latter case, MSCs adopt a neural fate in co-culture only after expressing nestin, an intermediate filament protein which is predominantly expressed during early stages of development by stem and progenitor cells of the central nervous system [3-6]. In cultivated MSCs, nestin expression requires serum-free conditions and at least 25 cell population doublings (corresponding to 10 cell passagings) to occur, suggesting a kind of maturation and/or selection of rat nestin-positive neurogenic MSCs [5].

During development, the CNS of vertebrates contains an abundant cell type designated as radial glial cells (RG). These cells while classically known as guiding cues for migrating neurons [7], play a major role as precursor cells for pyramidal neurons and astrocytes [8-11]. Since RG express a number of astroglial characteristics and can differentiate into astrocytes after completing their guidance function, they have been previously considered as integral part of the glial lineage. However, it was recently demonstrated that RG may persist as neural stem cells in several discrete regions of the adult brain. RG are expressing nestin both *in vivo *and *in vitro *[12] as well as an antigen related to nestin and recognized by the RC2 antibody [13]. While several growth factors and activities are known to act on the proliferation and differentiation of RG [7], their phenotype is also controlled *in vitro *by factors contained in serum [13]. Indeed, the transition between RC2-positive RG and stellate GFAP-positive astrocytes and its reversal can be induced, respectively, by withdrawal and addition of serum in the growth medium of cultures of developing cerebellar glial cells. In the case of MSCs, it is the withdrawal of serum that allows their expression of nestin suggesting that some serum-derived factors inhibit nestin expression in these cells.

Because serum-dependent effects on the stellation of astrocytes have been attributed to thrombin which is a normal serum component [14], we addressed the question of the regulation by thrombin of nestin expression in both MSCs and RG. Indeed there are indications that thrombin can have a regulatory role in nestin expression *in vivo *after experimental intracerebral haemorrhage [15]. We demonstrate that the presence of thrombin in the culture medium stimulates the growth of both RG and MSCs in a dose-dependent manner. In addition to the mitogenic effect, we demonstrate that thrombin represses nestin expression by MSCs but increases its expression by RG. We found that the PAR-1 receptor is involved in the regulation of nestin expression by both cell types to thrombin. Finally, we demonstrate that the modulation of nestin-expression by MSCs in serum-free conditions is dependent on their cell density.

## Results

### Serum and thrombin mitogenic effect on Radial Glia and Mesenchymal stem cells

To measure the effect of serum and thrombin on the proliferation of MSCs and RG, cells that had been maintained in serum-free conditions were allowed to incorporate BrdU in the presence of serum or thrombin (Table 1). An increase of the BrdU labelling index was observed for both MSCs and RG that were treated with serum or thrombin. While RG did not incorporate BrdU in the absence of serum, addition of thrombin at 2 nM stimulated their BrdU incorporation to 28% of that induced by serum. With MSCs, about 80% of all cells were incorporating BrdU in serum-free medium and addition of either serum or 900 pM thrombin to the medium lead to similar increases of labelling indexes up to 88 and 90% of all cells.

**Table 1:** Effect of serum and Thrombin on the proliferation of radial glia and rat MSCs

	- serum	+ serum	+ Thrombin
RG (n = 3)	1.8 +/- 1.62	43.73 +/- 3.95 (**)	12.2 +/- 3.22 (*)
MSCs (P10): all cells (n = 3)	79.9 +/- 0.35	88.5 +/- 0.23 (**)	90.4 +/- 2.29 (**)
Nestin-negative MSCs (n = 3)	68.8 +/- 0.74	84.7 +/- 1.54 (**)	83.2 +/- 2.21 (**)
Nestin-positive MSCs (n = 3)	86.0 +/- 4.9	92.0 +/- 8.9 (ns)	89.6 +/- 1.06 (ns)

Ns: no significant difference.

*: p < 0,05.

** p < 0,01.

RG and rat MSCs were cultured for respectively 2 and 3 days in the absence of serum and then were grown for two further days in the presence of serum or with 2 nM (RG) or 900 pM (rat MSCs) of thrombin. BrdU (20 μM) was added for the last 18 hrs before fixation with paraformaldehyde and immunofluorescent staining for both nestin and BrdU incorporation. Cells were counted on 10 microscopic fields (40×) on at least three coverslips in each condition. Percentage of BrdU-incorporating cells and standard deviation in each condition, obtained with GraphPad Prism4 Software, are shown. Levels of significance compared to the control (- serum) condition are shown.

We used a double immunostaining procedure for nestin and BrdU to analyze separately the BrdU incorporation by both nestin-positive and nestin-negative MSCs (Table 1). We found that 86% of nestin- positive MSCs incorporated BrdU and that this percentage did not change significantly after addition of serum or thrombin. Contrasting with this, we found that 68.8% of nestin-negative MSCs incorporated BrdU in the absence of serum and that this percentage increased significantly in the presence of thrombin and serum (p < 0.01, n = 3, 4000 counted cells) to reach values close to those of nestin-positive cells.

### Regulation of nestin expression by thrombin in Radial Glia and in Mesenchymal stem cells

**Figure 1:**
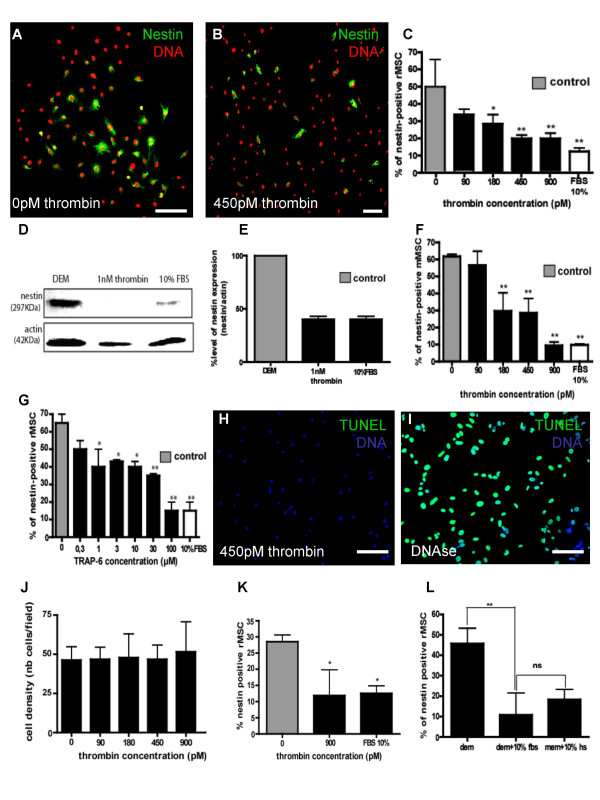
**Repression of nestin expression in rat MSCs cultures by serum, thrombin and TRAP6**. Rat MSCs and mouse MSCs of at least 25 population doublings were cultured for 3 days in serum-free medium to induce nestin expression. Then, the cells were renewed in DEM supplemented with different concentrations of thrombin (0 (A, rat MSCs), 90, 180, 450 (B, rat MSCs) and 900 pM), in DEM + 10% FBS or MEM + 10% horse serum (L) or in DEM + 900 pM of thrombin with 10^-5^M of FUdR (K) for a further 3 days period and then fixed and labelled with anti-nestin antibody (green). Nuclei were counterstained with TOPRO-3 (red) and then counted. In these conditions, there is a significant decrease of nestin expression both in rat MSCs (C) with 180 pM (*, p < 0,05), 450 and 900 pM of thrombin (**, p < 0,01) and in mouse MSCs (F) from 180 pM of thrombin (**). (L) There is however no difference when HS in MEM (18% of cell expressing nestin) is used instead of FBS in DEM (10% of cell expressing nestin). These results were confirmed by Western blotting (D), where nestin expression in rat MSCs cultivated in DEM, DEM + 1 nM of thrombin and DEM + 10% FBS was compared after 3 days of induction of nestin expression. The actin immunostaining controls the amount of protein loaded on the gel in each condition and allows to calculate the ratio between the intensity of the nestin and actin signals and to normalize data to the level of nestin expression in rat MSCs treated with DEM only (E). Rat MSCs were cultured for 3 days in serum-free medium to induce nestin expression. Then, those cells were renewed in DEM supplemented with different concentration of TRAP6, for a further 3 days period and then fixed and labelled with nestin antibody. Nuclei counterstained with TOPRO-3 and nestin-positive MSCs were then counted. (G) In these conditions, there is a significant decrease of nestin expression from 1 μM TRAP6 (*, p < 0,05; **, p < 0,01 from 30 μM, n = 3, ANOVA1). (H) To measure the effect of thrombin on the apoptosis of rat MSCs, cells were cultivated for 3 days in serum-free medium to induce nestin expression. Then, those cells were renewed in DEM with various concentrations of thrombin. After a new 3 days period, cells were fixed and submitted to a TUNEL labelling (green). Cell nuclei were counterstained with DAPI. For the positive control of the TUNEL assay, rat MSCs previously treated with DNAse were used (I). (J) To measure the effect of thrombin on cell density, cells were treated with the indicated concentrations of thrombin, then fixed, labelled with DAPI and counted in 10 microscopic fields at 10× magnification. Scale bar in A, B, H, I = 40 μm.

**Figure 2:**
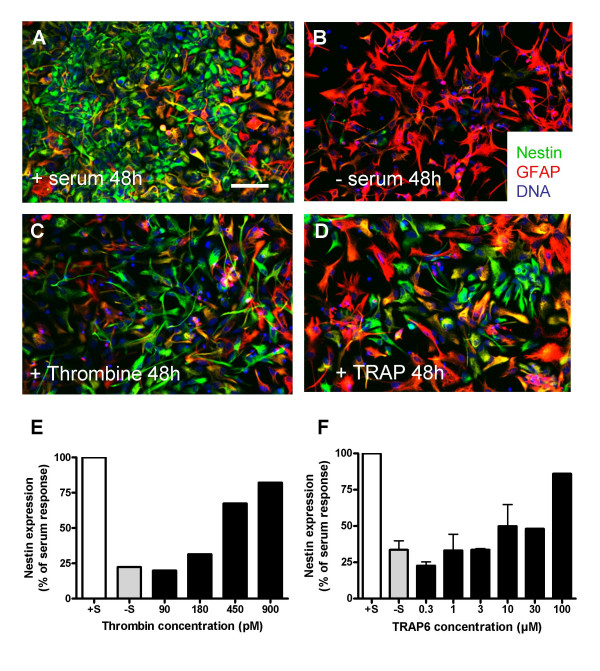
**Stimulation of nestin expression in RG cultures by thrombin and TRAP6**. RG were cultured for 2 days in the presence of serum (A) or were maintained for the same time in serum-free medium to suppress their nestin expression (B), or in serum-free medium plus 900 pM Thrombin (C) or 100 μM TRAP6 (D). Then, the cells were fixed and labelled with anti-nestin (green) and anti-GFAP (red) antibody. Nuclei were counterstained with TOPRO-3 (blue). In the presence of thrombin or TRAP6, the expression of nestin is maintained together with that of GFAP which is co-expressed in a varying proportion of the cells. Additional cell cultures that were treated in parallel with varying concentrations of thrombin (90 to 900 pM, E) or TRAP6 (0.3 to 100 μM, F) were collected and total protein extracts were prepared. Nestin concentrations in those extracts was then measured by Western blots as described in Material and Methods. Scale bar (in A = 40 μm) applies to all images.

MSCs and mouse RG production of nestin was assessed by immunostaining, leading to the conclusion that thrombin regulates nestin expression differently in the two cell types (Figure 1 and 2). A decrease in the number of nestin-positive MSCs was observed when, after 72 hours in serum-free culture medium allowing them to express nestin, they were grown further for 3 days in the presence of serum or thrombin (Fig. 1A–C). This effect of thrombin was dose-dependent with half-inhibition occurring at 180 pM thrombin (Fig. 1C).

TRAP is a hexapeptide that corresponds to amino acids 42 to 47 of the thrombin receptor and mimics the effect of thrombin independently of receptor cleavage [16]. When MSCs were incubated in DEM supplemented with different concentrations of TRAP6, a significant decrease of nestin-positive MSCs number occurred at concentration of TRAP6 equal to or above 1 μM (*, p < 0.05, Fig. 1G).

Those immunocytofluorescence observations were confirmed by a Western blotting approach. In immunoblots, the signal for nestin was observed in each condition but decreased in the presence of serum or thrombin while the signal obtained for actin remained unchanged (Fig. 1D, E).

In order to exclude an apoptotic effect of thrombin on MSCs that would lead to a specific decrease of the number of nestin-positive cells, we performed a TUNEL assay on MSCs treated with different concentrations of thrombin. No apoptotic cells were found in any condition, while a positive control, in which MSCs were treated by DNAse, showed a strong TUNEL labelling (Fig 1H,I).

An indirect effect of thrombin that would regulate nestin expression through an increase in cell density and cell to cell interactions can also be excluded. Although thrombin induces a proliferative effect on rMSC, no change in cell density occurred in cultures treated with increasing concentrations of thrombin for 3 days (Fig 1J). When, FUdR (10^-5 ^M) was added to inhibit DNA synthesis and cell proliferation, a significant decrease of nestin expression by rMSC cultivated in DEM + 900 pM thrombin or 10% serum was still observed indicating that thrombin inhibits directly nestin expression by rMSC (Fig 1K).

An opposite effect was seen with radial glial cells which stop expressing nestin after 48 hours without serum but maintain this expression in the presence of serum, thrombin or TRAP-6 (Fig. 2A–D). No such effect was seen on GFAP expression which was not investigated further. Western-blot analysis allowed to estimate at respectively 300 pM and 10 μM the concentrations of thrombin and TRAP-6 causing a half-maximal stimulation of nestin expression (Fig. 2E,F).

Because MSCs and RG were obtained from different rodent species and are usually grown in the presence of different sera, the effect of horse serum instead of bovine serum was tested on rat MSCs and the same experiment was repeated with mouse MSCs. No significant difference was found when nestin-positive rat MSCs were treated with DEM+10% FBS and MEM+10%HS, the medium used for RG cultivation (Fig. 1L), suggesting that FBS and HS exert the same effect on nestin expression of rat MSCs. A similar effect was observed when mouse MSCs (after 25 cell population doublings) were tested (Fig 1E), suggesting that the species difference between rat MSCs and mouse RG is not responsible for the observed different regulation of nestin expression by serum and thrombin.

### Thrombin receptor in Radial Glia and Mesenchymal Stem Cells

**Figure 3:**
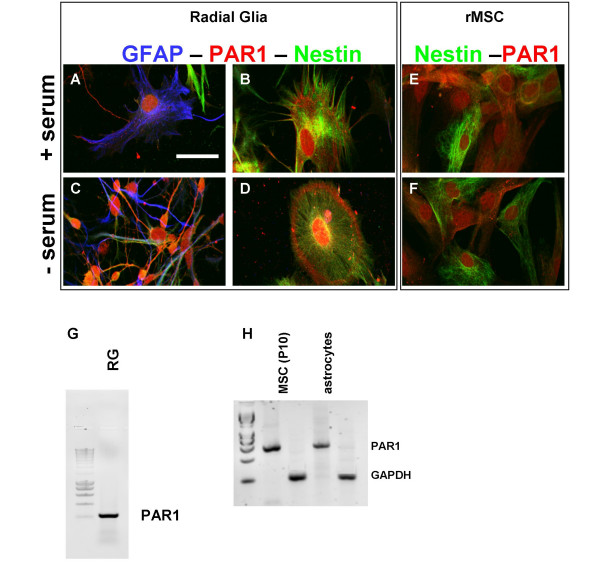
**Expression of the PAR1 thrombin receptor in RG and rat MSCs cultures**. RG and rat MSCs were cultured for 2 days in the presence of serum (A, B, E) or were maintained for the same time in serum-free medium (C, D, F). Then, the cells were fixed and RG were labelled with anti-nestin (green), anti-PAR1 (red) and anti-GFAP (blue) antibody (panel A-D) while rat MSCs were stained with anti-nestin (green) and anti-PAR1 (red) antibody (panel E, F). Scale bar (in A = 10 μm) applies to all images. Expression of thrombin receptor PAR1mRNA in mouse RG (G) and rat MSCs and astrocytes (H) was analyzed by RT-PCR using optimized primers for each species.

The presence of the specific Thrombin receptor PAR-1 was demonstrated on both RG cells and MSCs by immunocytochemistry using the H-111 antibody. In both cell types PAR-1 is expressed by cells grown in the presence or absence of serum (Fig. 3). The distribution of PAR-1 is indicative of a plasma membrane localization as it extends frequently to cell processes that the intermediate filaments Nestin or GFAP did not reach (Fig. 3A,B). More intense staining was also visible in the nucleus.

In MSCs, we observed by double-immunocytochemistry, the expression of PAR1 on both nestin-negative and nestin-positive MSCs suggesting that both cell types might be sensitive to the effect of thrombin (Fig. 3E–F).

To confirm the expression of PAR1 by MSCs and RG cells, RT-PCR analysis was performed on total RNA extracts of both cell types. Using primers optimized for rat and mouse transcripts, PAR1 mRNA was detected in mouse RG (Fig 3G) and in MSCs (Fig. 3H) at a level comparable to that of astrocytes of rat used as positive control. GAPDH mRNA served as internal control of the RT-PCR.

### Regulation of nestin expression by density of Mesenchymal stem cells

**Figure 4:**
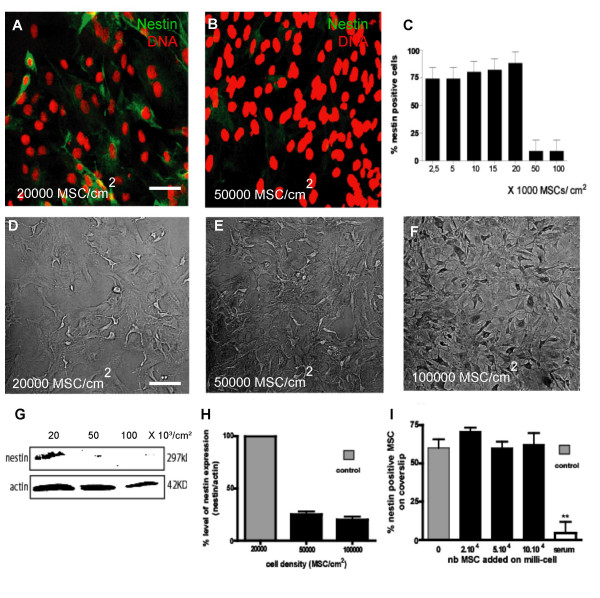
**Cell density regulation of nestin expression by rat MSCs in culture**. Rat MSCs were seeded at different cell densities (2.5 to 100·10^3 ^rMSCs/cm^2^) and maintained during 3 days in serum-free medium. These cells were then fixed, labelled with anti-nestin antibody (green), counterstained with TOPRO-3 (red) and positive cells were counted in triplicate cultures. A and B show typical results of anti-nestin immunostaining in culture seeded at initial densities of 20 and 50·10^3 ^cells/cm^2^. From 50000 rMSC/cm^2^, nestin expression decreases rapidly (C). In this experiment, results are expressed as percentages of nestin-positive cells in each culture conditions (n = 3, ANOVA1). These results were confirmed by Western blotting analysis. Rat MSCs were plated during 3 days in serum-free medium at different densities (20 (D), 50 (E) or 100 (F)·10^3 ^cells/cm^2^) and representative images were taken with a phase contrast microscope. Protein extracts were separated and revealed with anti-nestin and anti-actin antibodies. A decrease of nestin expression is apparent from an initial density of 50·10^3 ^cells/cm^2 ^(G). Actin immunostaining controls the protein amounts loaded on the gel in each condition (H). Rat MSCs were cultivated at a density of 10,000 rat MSCs/cm^2 ^together with different densities of rat MSCs in a physically separated millicell co-culture device. No significant difference was observed when the proportion of rat MSCs immunolabelled for nestin was compared in each condition (I) (n = 3, ANOVA1). Serum added in a millicell device as a positive control strongly inhibits nestin expression by rMSCs in the same setup. Scale bar A, B, D, E, F = 40 μm.

We have noticed that the effect of omitting serum in cultures of MSCs in order to increase their expression of nestin is variable in efficacy and that this variation could be a consequence of differing densities of MSCs stimulated by serum withdrawal. In order to determine how cell density could regulate the expression of nestin by MSCs, cells were plated at various densities in growth medium without serum for 3 days to induce nestin expression (Fig. 4A, B). We observed that at a density of 50,000 cells/cm^2 ^or above, MSCs decrease by more than 8-fold their nestin expression in response to serum withdrawal (Fig. 4C). Those results were confirmed by Western blot on cultures established at a density of 20, 50 or 100·10^3 ^MSCs/cm^2 ^which reached about 70, 90 and 100% confluence before extraction as shown by phase contrast microscopy (Fig 4D–F). Using identical amounts of total protein extracted from MSCs plated in serum-free conditions, nestin signal decreases with increasing cellular density (Fig. 4G). Quantification of the nestin expression in each condition was determined by calculating the ratio between the intensity of the nestin bands and the corresponding actin signals used as internal control for the protein contents loaded on the gel. This was normalized to the level of nestin expression in MSCs seeded at 20·10^3 ^cells/cm^2 ^(Fig 4H).

To further characterize the mechanism (soluble factor versus membrane-bound factor) responsible for such an effect, we co-cultivated MSCs coated on coverslips at a permissive density for 48 hours in serum-free medium together with different densities of MSCs physically separated in a millicell device (Fig. 4I). No significant decrease in nestin expression is observed in the MSCs grown at a permissive density on coverslips (n = 3, ANOVA-1 test, p > 0.05) in the presence of a high density of MSCs in a millicell. This clearly suggests that the decrease of nestin expression at high MSCs cell density requires a direct cell-to-cell contact. As a positive control of the permeability of the millicell device in such setup, MSCs were cultivated at a permissive density in serum-free medium with 30% FBS loaded into a millicell device. A strong decrease of nestin expression was indeed observed under this condition.

## Discussion

Thrombin, a multifunctional serine protease, plays central functions in haemostasis but also promotes a wide range of cellular responses via an interaction with specific seven-transmembrane domains receptors [17]. Thrombin and perhaps other coagulant proteases, mediates proliferative responses triggered by tissue damage [18-20]. *In vitro*, thrombin is the most potent activator of blood platelets [21,22], is chemotactic for monocytes [21,23], and is a potent mitogenic factor for vascular smooth-muscle cells[24], fibroblasts [21,25] and vascular endothelial cells [26,27]. Thrombin signalling is mediated by a family of G protein-coupled protease-activated receptors (PARs), for which PAR1 is the prototype [17]. Thrombin activates PAR1 through binding to and subsequent cleavage of the N-terminal domain of the receptor to expose a new amino-terminus that then acts as a tethered ligand to initiate intracellular signalling by the receptor [17]. We found on both radial glial cells and rat MSCs a membranous expression of PAR1 suggesting that thrombin may act on these cells physiology. We also found that PAR1 is expressed in the nucleus and cytoplasm. The intracellular staining for PAR1 can be explained by the presence of intracellular store of PAR1 in the cells [28].

There is increasing evidence suggesting that members of the serine protease family, including thrombin, chymotrypsin, urokinase plasminogen activator, and kallikrein, may play a role in normal development and/or pathology of the nervous system. Thrombin-like proteases have been shown to exert deleterious effects on different neuronal and non-neuronal cell populations *in vitro*, including neurite retraction and death [29-31]. Moreover, the proteolytic activity of thrombin has been shown to inhibit long-term morphological differentiation in serum-free cultures of several cell types, including spinal cord and brain neurons, neuroblastoma cells, astroglial and neuroepithelial cells [32-35].

Nestin expression in the adult nervous system has been detected in a number of pathological conditions including cerebral ischemia [36-38]., and traumatic brain injury [39]. Nakamura *et al.*[15] have demonstrated that intracerebral injections of low doses of thrombin that induces neuroprotection without causing detectable brain injury resulted in a marked increase in nestin expression indicating that this could constitute a protective mechanism induced in astrocytes by levels of stress that do not produce a definable lesion. Nestin expression could, therefore, represent an embryonic reversion of the mature cytoskeleton that may aid in the response to and recovery from a wide variety of cerebral injuries, but might also perhaps aid in damage prevention.

In this study, we demonstrate that nestin expression is regulated by thrombin in opposite manner in RG and MSCs although the proliferation of both cell types is stimulated by this protease. Thrombin effect is thus associated in both cell types with a proliferating, undifferentiated state but in RG cells this involves the induction of nestin expression, a marker of immaturity for neural progenitors. In MSCs however, nestin expression corresponds to the first step toward acquisition of a neural fate and thus corresponds to a progression from the mesenchymal "undifferentiated", proliferating phenotype [3-5]. Maintenance of MSCs proliferation in the presence of thrombin would indeed be associated with an inhibition of nestin expression by MSCs as it constitutes one feature of their undifferentiated state. A possible selection of one population type over the other can be discounted as an explanation for the decrease of nestin expression by MSCs in the presence of thrombin because this effect still occurs in cells that cannot proliferate when treated with FUdR. Furthermore, the absence of apoptotic cells in the cultures treated with thrombin as well as the similar proliferation rates for both nestin-negative and nestin-positive MSCs which are induced in the presence of serum or thrombin in the growth medium demonstrate that thrombin represses directly the expression of nestin by MSCs and does not act by selecting the population of nestin-negative cells.

Thus unlike radial glial cells, MSCs *in vitro *respond to thrombin by a decrease of nestin expression. The nestin gene contains two tissue-specific transcriptional elements : a cis-element in the first intron drives nestin expression in somitic muscle precursors, while an enhancer in the second intron directs expression to CNS precursor cells [40] and thus to radial glial cells. Which of these two regulatory elements controls nestin expression by MSCs remains to be determined.

Finally, we demonstrate that nestin expression by MSCs decreases at a high cell density, as observed by immunocytofluorescence and Western blot assay. Tropepe *et al.*[41] have found that embryonic stem cells express nestin when they differentiate into neural cells. This expression and the subsequent neural differentiation of embryonic stem cells are inhibited by a high cell density suggesting a similar behaviour for both MSCs and embryonic stem cells. It has also been demonstrated that a high cell density could accelerate the differentiation of human bone marrow MSCs into chondrocyte in a chondrogenic differentiation medium [42]. However, this chondrogenic differentiation is mediated by soluble factors while the decrease of nestin expression observed in our experiments is mediated by a direct cell-to-cell contact. Since our experiments were made with MSCs obtained after more than 10 passages, we cannot exclude that these apparently different regulatory processes for the high cell density effect on MSCs mesodermal fate result from the use of MSCs of early and late passages.

There are however indications that MSCs of late passage secrete a soluble factor that can act on other cell types. Indeed Wislet-Gendebien *et al.*[43] have already demonstrated that nestin-positive MSCs secrete BMP4, a member of TGFβ family, which stimulates the astrocytic differentiation of co-cultured neural stem cells, a maturation process that involves a loss of nestin expression by these cells. However, our co-culture experiments did not show any significant decrease in the proportion of nestin-positive MSCs when cells at a permissive density were grown with other MSCs at higher densities in a setup that allows no direct contact, suggesting rather a requirement for a direct cell-to-cell contact for nestin repression. Alternatively, high cellular density by increasing the direct cell-to-cell contact in the culture dish could inhibit specifically the proliferation of nestin-positive MSCs which then could explain their decrease in number as the overall cellular density increases.

During the last few years, a number of studies have addressed the phenotypic plasticity of MSCs. Most of these studies were performed *in vivo *and demonstrated that environmental factors play important roles in determining the ability of grafted MSCs to adopt a neural-like phenotype. Nakano et al. showed that murine bone marrow cells adopt a neural fate when they are directly injected into the striatum of previously irradiated mice [44]. Similarly, MSCs also adopt neural fate after systemic injection in lethally irradiated mice [1]. Interestingly, the systemic injection of MSCs in non-irradiated but brain-lesioned mice had positive effects on injury repair, but very few MSCs-derived cells expressed neural marker in such conditions.

## Conclusion

This work shows that the presence of thrombin in the culture medium stimulates the growth of both RG and MSCs and that nestin expression by MSCs and RG can be regulated in opposite manner by thrombin *in vitro*. Thrombin effect is thus associated in both cell types with a proliferating, undifferentiated state but in RG this involves the induction of nestin expression, a marker of immaturity for neural progenitors. In MSCs however, nestin expression, as it corresponds to a progression from the mesenchymal "undifferentiated", proliferating phenotype toward acquisition of a neural fate, is inhibited by the mitogenic signal. As we know that thrombin is expressed in the central nervous system [45] and is present in higher concentration during brain injury and implicated in the response to brain lesions [15], it will be interesting to study the effect of thrombin on the regulation of the neural phenotypic plasticity of MSCs *in vivo *in the context of their proposed use as grafting material for cell therapeutic approaches of the central nervous system lesions.

## Methods

### Preparation and culture of rat mesenchymal stem cells (MSCs)

Tissue used for preparing the cell cultures was obtained from animals bred and kept in the University central animal facility in accordance with the rules set by the local animal ethics committee. Adult rat MSCs were obtained and cultivated as previously described [3,4,43]. After 25 cell population doublings in DEM/10% FBS (Invitrogen^®^, Belgium), nestin expression was initiated by washing the cultures with PBS and then growing them in DEM/F12 medium (Invitrogen^®^) at various densities (between 2,500 and 100,000 MSCs/cm^2^) on coverslips or in a T75-Flask. Adult mouse bone marrow was obtained from femur and tibias by aspiration and was resuspended into 5 ml of DEM (Invitrogen^®^, Merelbeke, Belgium). About 10 × 10^7 ^marrow cells were plated on 25-cm^2 ^tissue culture flask in MesenCult Basal Media supplemented with MSC stimulatory supplements (StemCell Technologies^®^, Vancouver, Canada). After 24 hours, the non-adherent cells were removed by replacing the medium. When the mMSC became confluent, they were resuspended with 0,25% trypsin and 1 mM EDTA and then sub-cultured. To initiate nestin expression, the culture were washed with PBS and grown in DEM/F12 medium (Invitrogen^®^). After induction of nestin expression for 3 days, MSC plated at a density of 20,000 MSC/cm^2 ^were transferred into DEM supplemented with either 10% Foetal bovine serum (FBS), different concentrations of thrombin (Calbiochem^®^, cat 605157, Belgium) or of TRAP6 (Bachem^®^, H-8365, Belgium), 10^-5 ^M FUdR (Sigma) or into MEM with 10% of horse serum. After 3 days, the cells on coverslips were fixed or total proteins were extracted from the T75-Flask. MSCs were then processed for immunocytofluorescence or Western blotting as described below.

### Preparation and culture of Radial Glia (RG)

NMRI mice embryos were used. The day of conception was determined by the presence of a vaginal plug (embryonic day 0). Dissociated cerebellar cells culture were prepared from E16 or E17 embryos by digestion of minced cerebella with 0.25% trypsin and 0.01% DNase 1 (Sigma^®^, Belgium) for 22 min at 37°C, followed by trituration in a flamed Pasteur pipette and filtration through a 40 μm nylon mesh. Cells were then seeded as 50-μl drops containing 125,000 cells on glass coverslips coated with 10 mg/ml polyornithine (Sigma^®^). After 30 min, the cells were washed once with cold PBS to remove loosely adhering neurons and 500 μl of growth medium (MEM containing 6 g/l glucose and 10% horse serum Invitrogen^®^) was added. This medium was changed every 2 days. For experiments, RG cells were incubated in serum-free medium during 48 hours and then treated in MEM supplemented with serum or different concentration of thrombin or TRAP6.

### Immunocytofluorescence

The cultures were fixed with 4% (v/v) paraformaldehyde for 15 minutes at room temperature and washed 3 times in Tris-buffered saline (TBS) buffer. They were then permeabilized in 1% Triton-X100 (v/v) for 15 minutes and washed 3 times in TBS buffer. Non-specific binding was blocked by a 1 hour treatment in TBST (TBS buffer with 0.1% Tween-20) containing defatted milk powder (30 mg/ml). The cells were then incubated for 1 hour at room temperature with anti-nestin (Rat401, mouse IgG, Pharmingen^®^, Belgium, dilution 1:1500), anti-GFAP (Mouse or rabbit IgG, Dako^®^, dilution 1:500) or anti-PAR1 (H-111, rabbit IgG, BD Biosciences^®^, Belgium, dilution 1:250) diluted in blocking buffer. After 3 TBST washes, cells were incubated in FITC- or Cy5-conjugated anti-mouse IgG (Jackson Immunoresearch^®^, Belgium, 1:500) or rhodamine-conjugated anti-rabbit IgG (Jackson Immunoresearch^®^,1:500) for 1 hour at room temperature and in the dark. The nuclei were stained with 5 μM TOPRO-3 iodide (Molecular Probes^®^, Belgium) and mounted in Fluoprep (Biomerieux^®^, France) or with vectashield hard set mounting medium with DAPI (Vector laboratories^®^, Belgium). The preparations were then observed using a Bio-Rad MRC1024 laser scanning confocal microscope.

### TUNEL assay

The cultures were fixed with 4% (v/v) paraformaldehyde for 15 minutes at room temperature and washed 3 times in Tris-buffered saline (TBS) buffer. They were then permeabilized in 1% Triton-X100 (v/v) with 0,1% sodium citrate for 15 minutes and washed 3 times in TBS buffer. Then, the presence of apoptotic cells was assayed with the TUNEL label mix (Roche applied Science^®^, Belgium) following the manufacturer instructions.

### Western Blot

Total protein extracts were obtained from confluent cells that had been cultured in the different media. The cells were harvested by scraping the dish in 500 μl lysis buffer (0,6 M KCl, 5 mM EGTA, 5 mM EDTA, 1% Triton-X100 and 1 mM PMSF in PBS). The cells were then fractioned into pelletable (insoluble) and non-pelletable (soluble) proteins by centrifugation at 30,000 × g for 15 minutes at 4°C. The pellet was suspended in electrophoresis loading buffer (glycerol 20% v/v; SDS 2%; Tris 0,1 M pH 6,8 ; 5% v/v β-mercaptoethanol; bromophenol blue) and the suspension was centrifuged at 30,000 × g for 15 minutes at 4°C. The supernatant was used for protein concentration measurement using the "RC DC Protein Assay" (Bio-Rad^®^, Belgium). After normalization of protein contents, the extracts were separated by electrophoresis on Phastgel 4–15% SDS gradient using the Phast System Instrument (GE Healthcare^®^, Netherlands) and transferred to a PVDF membrane using 25 mM Tris, 192 mM glycine and 20% (V/V) methanol as blotting buffer. The membranes were saturated with 3% gelatin (BioRad^®^), incubated for 1 hour with the monoclonal antibody against rat nestin (Pharmingen^®^, 1:1,000) or β-actin, (Sigma^®^, 1:5,000) at room temperature and then washed several times with TBST. The membrane was then incubated in biotinylated goat anti-mouse antibody (Roche^®^, Brussels, Belgium; 1:5,000) for 1 h at room temperature. After several washes in TBST, the membrane was incubated with peroxidase-coupled streptavidin (1:100,000, Sigma^®^) at 37°C during 1 h. Signals were visualized with a solution of 0.01% sodium perborate in 50 mM phosphate buffer containing 2% dioctyl sulfosuccinate (40 mg in 1 ml of dimethylformamide) and 2% 3-3'-5-5'-tetramethyl benzidine (20 mg in 1 ml of dimethylformamide). Images were acquired with the Magic Scan program and band intensities were evaluated with Image Master 1D Elite.

### Reverse transcription-polymerase chain reaction (RT-PCR) analysis

Total RNA of rat MSCs and mouse RG cells were isolated with the RNeasy Midi Kit (Qiagen^®^, Belgium) as described in the protocol of the manufacturer, and treated with RNAse-free DNAse to remove any possible contamination by genomic DNA. As determined by 260/280 OD readings, 1 μg of total RNA was reverse-transcribed using primers with oligodT and 200 U of reverse transcriptase (Superscript, Invitrogen^®^). A 2 μl aliquot of the resulting cDNA reaction, used as template, was added to a 50 μl PCR reaction mixture containing: 0.2 μM of both forward and reverse primers synthesized by Eurogentec^® ^(Belgium), 0.2 mM of each dNTP, 1.5 mM of MgCl_2 _and 5 U of Taq polymerase (Promega^®^, The Netherlands). The PCR program was achieved in a PTC 200 instrument (MJ Research^®^, USA). After a 2 min denaturation step at 94°C, amplifications were carried out for 35 cycles (94°C for 1 min, 55°C for 1 min and 72°C for 1 min), followed by a final extension at 72°C for 1 min. Ten microlitres of the PCR reaction was analysed in a 3% agarose gel in tris-acetic acid-EDTA (TAE) buffer. The primers used in PCR reaction for PAR1 are 5'-TGA CAG TCA TAA GCA TTG AC-3' for the rat forward primer and 5'-GGC ATA GTA GTA AAT CAA GG-3' for the rat reverse primer, 5'-TAT-CCG-ATC-CAG-TCC-CTG-TC-3' for the mouse forward primer and 5'-AGA-CCG-TGG-AAA-CGA-TCA-AC-3' for the mouse reverse primer. The annealing temperature for mouse PAR1 PCR was set at 58°C. For rat GAPDH, the primers used in PCR reaction were 5'-GAC CCC TTC ATT GAC CTC AAC TAC ATG-3' for the forward primer and 5'-GCC TTC TCC ATG GTG GTG AAC AC-3' for the reverse primer.

### BrdU incorporation assay

After 3 days of culture in serum free medium, bromodeoxyuridine (BrdU) (20 μM, Sigma^®^) was added to RG and MSCs cultures (cultivated in serum- or thrombin- containing medium) for 18 hours before fixation with paraformaldehyde and immunofluorescent staining. For BrdU labelling after nestin immunolabelling performed as described above, coverslips were post-fixed for 10 minutes in 4% (v:v) paraformaldehyde, incubated in HCl 1 N for 20 minutes at 37°C, washed with sodium borate solution (50 mM, pH 8.5) and finally incubated with anti-BrdU antibody for 1 hour at room temperature (Oxford Biotechnology^®^, rat IgG, dilution 1:200) and Cy-5-conjugated anti-rat antibody, 1 hour at room temperature. The preparations were observed as described above.

### Cell counting and statistical analysis

For each condition 2 coverslips per culture and 10 microscopic fields (40×)/coverslip were counted for three separate cultures. Statistical analyses were performed using an ANOVA-1 test and Dunnett post-test.

## Authors' contributions

FW carried out part of the experiments on mesenchymal stem cells and drafted the manuscript. SWG carried out part of the experiments on mesenchymal stem cells. GC carried out the experiments on radial glial cells. BR participated in the design and coordination of the study. PL conceived of the study, and participated in its design and coordination and helped to draft the manuscript. All authors read and approved the final manuscript.
